# 

**Published:** 1904-02-15

**Authors:** 


### ANNOUNCEMENTS.

---

THE next annual meeting of the American Dental Society of Europe will be held at the Hamburger Hof, Hamburg, Germany, April 1st to 4th, 1904. We request the publication of this announcement in your esteemed journal. Yours truly,

DR. CHARLES J. MONK, *Hon. Sec.*

---

THE Board of Dental Examiners of California will hold its next examination in San Francisco, commencing on May 23d, 1904, and will also hold an examination in Los Angeles, commencing on June 13th, 1904.

Respectfully yours,

F. G. BAIRD,

---

THE St. Louis Dental Society elected officers for the year at its monthly meeting held December 1, 1903. The new officers are: President, Herman Prinz; Vice-President, C. DeWitt Lukens; Secretary, James F. Austin; Treasurer, Joseph G. Pfaff; Librarian W. A. Roddy; Corresponding Secretary, DeCourcey Lindsely. The retiring president is T. E. Turner. Secretary J. F. Austin and Treasurer Joseph G. Pfaff were re-elected.

---

THE Michigan State Board of Examiners will hold its next semi-annual session May 10th, 1904, at Grand Rapids.

W. C. MCKINNEY, *Sec.*

Saginaw, Mich.

---

THE New Jersey State Dental Society will hold its annual convention in the Auditorium at Asbury Park, N. J.,

July 21st, 22d and 23d, next. The Exhibit Committee is prepared to allot space to exhibitors. Those desiring space please make application direct to the chairman.

DR. W. G. CHASE, Princeton, N. J.

---

THE National Association of Dental Examiners will hold its annual meeting in the Coliseum Building, corner 13th and Olive streets, St. Louis, Mo., on the 25th, 26th and 27th of August, beginning promptly at 10 A. M. Telephone and telegraph offices in building. Hotel accomodations will be secured for the members.

CHAS. A. MEEKER, *Sec. and Treas.*

